# On the Origin of Voltaic Action

**Published:** 1831

**Authors:** 


### II. ANALYTICAL.

1. *On the Origin of Voltaic Action.*—M. Matteuce, a celebrated French Chemist, contends, that the mere contact of different metals, is sufficient for the development of electricity, although he admits that chemical action exerts an influence over this force, just as heat does in thermo-electric experiments. In order to obviate every suspicion of chemical action, he washed a frog in distilled water, freed from air, and thus removed all animal fluid. It was then suspended by the nerves from the zinc wire, the jar filled with distilled water, and then with pure hydrogen; but on touching the limbs with the copper wire, the same contractions took place as in common air.

This question is now dividing the philosophers of Europe, and is certainly a very important one.—*Journ. of Royal Institution, May*, 1831.

